# Comparison of reference distributions acquired by direct and indirect sampling techniques: exemplified with the Pediatric Reference Interval in China (PRINCE) study

**DOI:** 10.1186/s12874-022-01596-8

**Published:** 2022-04-10

**Authors:** Ruohua Yan, Kun Li, Yaqi Lv, Yaguang Peng, Nicholas Van Halm-Lutterodt, Wenqi Song, Xiaoxia Peng, Xin Ni

**Affiliations:** 1 Center for Clinical Epidemiology and Evidence-Based Medicine, Beijing Children’s Hospital, Capital Medical University, National Center for Children Health, No.56 Nanlishi Road, Beijing, 100045 China; 2 Department of Orthopaedics and Neurosurgery, Keck Medical Center of USC, University of Southern California, Los Angeles, USA; 3 Department of Clinical Laboratory Center, Beijing Children’s Hospital, Capital Medical University, National Center for Children Health, Beijing, China; 4 Beijing Key Laboratory for Pediatric Diseases of Otolaryngology, Head and Neck Surgery, Beijing Children’s Hospital, Capital Medical University, National Center for Children Health, No. 56 Nanlishi Road, Beijing, 100045 China

**Keywords:** Reference distribution, Direct sampling techniques, Indirect sampling techniques, SOM, GMM

## Abstract

**Background:**

Our study aimed to compare the reference distributions of serum creatinine and urea obtained by direct sampling technique and two indirect sampling techniques including the Gaussian Mixture Model (GMM) and the Self-Organizing Map (SOM) clustering based on clinical laboratory records, so that the feasibility as well as the potential limitations of indirect sampling techniques could be clarified.

**Methods:**

The direct sampling technique was used in the Pediatric Reference Interval in China (PRINCE) study, in which 15,150 healthy volunteers aged 0 to 19 years were recruited from 11 provinces across China from January 2017 to December 2018. The indirect sampling techniques were used in the Laboratory Information System (LIS) database of Beijing Children’s Hospital, in which 164,710 outpatients were included for partitioning of potential healthy individuals by GMM or SOM from January to December 2016. The reference distributions of creatinine and urea that were established by the PRINCE study and the LIS database were compared.

**Results:**

The density curves of creatinine and urea based on the PRINCE data and the GMM and SOM partitioned LIS data showed a large overlap. However, deviations were found in reference intervals among the three populations.

**Conclusions:**

Both GMM and SOM can identify potential healthy individuals from the LIS data. The performance of GMM is consistent and stable. However, GMM relies on Gaussian fitting, and thus is not suitable for skewed data. SOM is applicable for high-dimensional data, and is adaptable to data distribution. But it is susceptible to sample size and outlier detection strategy.

**Supplementary Information:**

The online version contains supplementary material available at 10.1186/s12874-022-01596-8.

## Background

Reference interval (RI) is one of the most widely used decision-making tools in clinical practice [1]. Pediatric RIs are critical for not only the diagnosis and treatment of diseases for sick children, but also the presentation of physiological conditions for healthy children during growth and development. We have recently reported a critical gap of pediatric RIs in China, which suggests the necessity of establishment of RIs for Chinese children [2].

In general, directly sampling reference individuals from a well-defined “healthy population” is a classic approach to establish RIs [1]. Since 2016, a nationwide cross-sectional study named Pediatric Reference Intervals in China (RPINCE) has been conducted, aiming at depicting the reference distributions of laboratory indexes based on direct samples [3]. However, direct sampling technique is found to be challenging to enroll adequate samples of children due to ethical and feasibility reasons, particularly for special age groups such as neonates. Over the past years, several indirect techniques have been proposed, including the Gaussian Mixture Model (GMM) and the Self-Organizing Map (SOM) clustering, by which RIs are established based on normal values partitioned from routine medical records [4–6]. The main procedure of indirect sampling technique can be summarized as a subgroup division of potential healthy population from the overall population, which is also referred to as unsupervised clustering [7]. For instance, GMM is a good way to separate mutually overlapping clusters by describing the distribution of variables through multiple Gaussian probability density functions [8, 9]. Each density function represents a cluster, and the parameters of density functions are independent of each other. When the number of density functions is large enough, GMM can approach most objects with high precision. On the other hand, SOM clustering, as a kind of neural network model, can map data points to several grid structures, with each grid be spread out from the center point of a cluster [10]. SOM clustering can provide more intuitive results and are more suitable for processing complex data, and thus has achieved many successful applications in engineering field [11, 12]. However, SOM clustering has been rarely reported in biomedical field till now.

Although indirect sampling technique is simple and fast by obtaining laboratory indexes from the hospitals’ Laboratory Information System (LIS) database, whether it can replace direct technique is always controversial [13]. Therefore, it will be interesting to compare the reference distributions of laboratory indexes (taking serum creatinine and urea as examples) of potential healthy children partitioned from the LIS database by GMM or SOM with those of reference individuals in the PRINCE study. By this comparison, the feasibility as well as the potential limitations of indirect sampling techniques can be clarified.

## Methods

### Data sources

The PRINCE study is a typical example of direct sampling technique. It aims to establish and verify pediatric RIs based on 15,150 apparent healthy children recruited from 11 centers across China between January 2017 and December 2018. The eligibility criteria and other detailed information have been published in the study protocol [3]. In brief, blood specimens were phlebotomized by trained pediatric nurses using a BD Vacutainer and vacuum tube needles (Becton, Dickinson and Company, Dublin, Ireland). Specimens for biochemical markers were centrifuged (relative centrifugal force, 1200 g) for 10 min after clotting, followed by approximately 30 min’ quiescence at room temperature (22 to 25 ℃). The serum was divided into aliquots (minimum, 0.5 mL) using well-sealed freezing containers and stored at -80 ℃ within 8 h after collection. All aliquots were transported to the central laboratory at Beijing Children’s Hospital through cold chain. The measurement of samples was completed within 6 months after collection; repeated freeze–thaw cycles were avoided during the examination process. Serum creatinine and urea were measured using Roche Cobas C702 (Roche Diagnostics GmbH, Mannheim, Germany) with enzymatic method. The PRINCE study was approved by the Ethics Committee of Beijing Children’s Hospital and all participating centers.

Data used for indirect sampling techniques were derived from the LIS database of Beijing Children’s Hospital. All serum creatinine and urea measures of outpatients from January to December 2017 were extracted. Furthermore, to better evaluate the renal function of outpatients, uric acid was also obtained. Biochemical markers were tested by Beckman Coulter AU5800 (Beckman Coulter Inc., California, USA) within 2 h after venous blood sample collection. The Department of Clinical Laboratory Center of Beijing Children’s Hospital is certificated by ISO15189.

### Data cleaning

Data cleaning was performed for the PRINCE data and the LIS data, respectively. For the PRINCE data, children with unhealthy conditions such as taking medications within 1 week, having acute illness or fever within 2 weeks, receiving operation or blood transfusion within 1 month, or suffering from chronic illness or congenital disease were excluded. Furthermore, specimens that were failed to collect or of substandard quality (e.g., hemolysis) were removed. For the LIS data, missing and extreme values were firstly deleted from the database. Then repeatedly tested children were checked, with an assumption that the necessity of multiple testing implies higher chances of pathology [14]. If an individual had two or more laboratory records within a year, the earliest record would be utilized. Taking into account the adverse effect of insufficient sample size on the stability of cluster analysis, children aged less than 1 year or beyond 17 years were not included in the present study.

During the process of outlier detection, age and sex partitioning were performed by decision tree at first (Supplement Fig. 1) [15]. The decision tree of creatinine indicated an age partition of 1 to < 6 years, 6 to < 12 years, and 12 to < 17 years (Supplement Fig. 2). Since significant difference of creatinine was found between boys and girls after puberty (Supplement Fig. 1A), the subgroup of 12 to < 17 years was partitioned by sex as well. Contrary to creatinine, urea did not show substantial age and sex variation (Supplement Fig. 1B), and was not partitioned into subgroups in the present study. Subsequently, the normality of test results in each age and sex subgroup was checked, and Box-Cox transformation was used as appropriate to ensure that the data obeyed Gaussian distribution [16]. Box-Cox transformation could be expressed by the equation of y = (x^λ^-1)/λ (λ ≠ 0), where y represents transformed value of x using the power λ, and λ was estimated by maximum likelihood approach. Finally, Tukey method was used to detect outliers [17], in which outlying values were defined as less than Q1-1.5 × IQR or more than Q3 + 1.5 × IQR, where Q1 is the 25th percentage, Q3 is the 75th percentage, and IQR is the interquartile range (Q3-Q1). The flow chart of data cleaning is shown in Supplement Fig. 3.

### Transference between biochemistry analyzers

Since the biochemistry analyzers and reagents used by the PRINCE study varied from clinical routine, systematic errors might exist between measurements of the PRINCE data and the LIS data. Therefore, transference was made for the PRINCE data from Roche to Beckman, in order to provide more objective comparison of direct and indirect sampling techniques [1]. According to the published transference formula [18, 19], creatinine (enzymatic) of Abbott ARCHITECT c8000 was × 0.965–0.447 to transfer to Roche Cobas 6000, and was × 0.903–1.192 to transfer to Beckman Coulter AU Systems. On the other hand, urea of Abbott ARCHITECT c8000 was × 0.941 + 0.143 to transfer to Roche Cobas 6000, and was × 0.961 + 0.110 to transfer to Beckman Coulter AU Systems. As the results, the values of creatinine and urea in the transferred PRINCE data were calculated by (creatinine + 0.447) × 0.936–1.192 and (urea-0.143) × 1.021 + 0.110 in the PRINCE data, respectively.

### Indirect sampling techniques

GMM and SOM were used to partition potential healthy children from the LIS data. The GMM method was performed in *mixtools* package of R 3.5.3 (https://www.r-project.org) [20]. The main procedure of GMM was based on Expectation Maximum algorithm [8]. Three clusters were set to represent unhealthy (with low and high levels of analytes) and potential healthy individuals (with normal level of analytes). The starting distribution parameters of the three clusters referred to the transferred PRINCE data, i.e., μ were set as the lower, median, and upper quartiles of analytes in the transferred PRINCE data, and σ were set as the standard deviation (SD) of analytes in the transferred PRINCE data. By simulating the initial distributions of the clusters, which cluster each data point was more likely to come from could be decided, and the distribution parameters could be re-calculated after classifying all data points. The iteration was performed until the distribution parameters converged to an ideal state. Then potential healthy children could be identified through the final classification.

The SOM method was implemented by JMP 13.0.0 (https://www.jmp.com). Unlike GMM that screened reference values for each independent analyte, SOM could consider multiple related biochemical markers simultaneously through a network structure. Creatinine, urea, and uric acid were assigned as column variables and were scaled individually, and then SOM with three clusters was structured according to batch algorithm using a locally weighted linear smoother. The number of rows and columns in cluster grid were set as three and one, respectively, to represent children with low, normal, and high levels of renal function. The goodness-of-fit of SOM was evaluated by cubic clustering criterion. Procedures of SOM clustering were conducted as follows: (1) the initial center points were determined by principal component analysis; (2) a grid was laid out in each principal component space with 2.5 standard deviations’ edges from the middle in all directions; (3) each data point was assigned to the closest cluster; (4) the center points were re-estimated by cluster means, and the data points were re-classified to the closest clusters. The iteration was proceeded until convergence.

### Statistical analysis

The probability density diagrams for creatinine and urea were plotted by age and sex subgroups in the PRINCE data, the transferred PRINCE data, the LIS data, the GMM partitioned LIS data, and the SOM partitioned LIS data, respectively. Reference distributions of analytes acquired by direct and two indirect sampling techniques were graphically presented, and corresponding RIs were calculated by non-parametric method using MedCalc 15.10.0 (https://www.medcalc.org).

In view of the complexity of pediatric outpatients in the LIS data, a more radical outlier detection strategy was implemented, in order to explore the influence of outliers on the results of indirect sampling techniques [21]. In this strategy, children with an outlier in either creatinine, urea, or uric acid were excluded. Other statistical analyses were same as described above.

To assess the bias of RIs established by indirect sampling techniques from that established by direct technique, the ratio of between-method difference in reference limits to between-individual SD was calculated, where between-individual SD is 1/3.92 of RI width established by direct technique [22]. The conventional threshold of bias ratio is 0.25 (allowable) or 0.375 (minimal), which can be used to judge the performance of GMM and SOM.

## Results

### Original distributions of the PRINCE data and the LIS data

In total, 10,685 measures of creatinine and 10,663 measures of urea were included in the PRINCE data, while 123,105 measures of creatinine and 122,421 measures of urea were included in the LIS data (Supplement Fig. 3). The original distributions of creatinine and urea in the PRINCE data, the transferred PRINCE data, and the LIS data are shown in Table 1 and Supplement Fig. 4. From the probability density diagrams we noticed that, after transference, the systematic errors brought by different biochemistry assays were largely corrected, especially for creatinine. However, creatinine in the LIS data shaped more right-skewed than that in the PRINCE data (Supplement Fig. 4A-4D). Meanwhile, the distribution of urea in the PRINCE data and the LIS data were similar and both close to symmetrical (Supplement Fig. 4E).

**Table 1:** Original distributions of creatinine and urea in the PRINCE data and the LIS data

Age Group	PRINCE						Transferred PRINCE						LIS					
	**Boys**			**Girls**			**Boys**			**Girls**			**Boys**			**Girls**		
	**n**	**Mean**	**SD**	**n**	**Mean**	**SD**	**n**	**Mean**	**SD**	**n**	**Mean**	**SD**	**n**	**Mean**	**SD**	**n**	**Mean**	**SD**
**Creatinine (μmol/L)**																		
** 1 to < 2 years**	269	25.0	3.7	229	24.1	3.7	269	22.6	3.4	229	21.8	3.5	9137	25.27	6.48	6004	25.06	7.05
** 2 to < 3 years**	320	28.7	4.3	292	28.9	4.5	320	26.1	4.0	292	26.2	4.2	8431	27.34	7.02	5218	26.75	7.14
** 3 to < 4 years**	462	32.6	4.3	355	31.1	4.1	462	29.8	4.0	355	28.4	3.8	7783	29.79	7.57	5243	29.18	7.43
** 4 to < 5 years**	315	35.0	4.6	316	34.6	4.4	315	32.0	4.3	316	31.6	4.2	7102	31.85	7.59	4762	31.13	7.55
** 5 to < 6 years**	307	37.1	4.5	302	37.0	4.5	307	33.9	4.2	302	33.9	4.2	5856	33.57	7.97	3933	32.73	7.83
** 6 to < 7 years**	384	41.2	5.2	350	40.2	4.9	384	37.8	4.9	350	36.8	4.6	4847	37.18	8.10	3352	36.38	8.01
** 7 to < 8 years**	414	42.0	5.2	374	41.0	4.9	414	38.5	4.9	374	37.6	4.6	4590	38.94	8.60	3538	38.23	8.44
** 8 to < 9 years**	405	44.0	5.5	404	43.1	5.6	405	40.4	5.1	404	39.6	5.2	4769	40.98	9.35	3735	39.95	8.78
** 9 to < 10 years**	389	46.1	5.3	369	45.2	5.3	389	42.3	4.9	369	41.5	5.0	4115	41.82	9.35	3507	40.47	9.06
** 10 to < 11 years**	402	48.5	5.5	381	45.4	5.3	402	44.6	5.2	381	41.7	5.0	3568	43.74	9.60	2794	42.17	9.56
** 11 to < 12 years**	292	49.1	5.5	305	45.5	5.7	292	45.2	5.1	305	41.8	5.3	3419	46.25	9.72	2678	43.72	9.80
** 12 to < 13 years**	408	55.4	8.6	379	51.3	8.1	408	51.1	8.0	379	47.3	7.5	2718	49.18	11.24	2079	45.82	11.01
** 13 to < 14 years**	287	59.9	10.0	309	53.6	7.9	287	55.3	9.4	309	49.4	7.4	2418	53.81	13.06	1957	48.65	11.55
** 14 to < 15 years**	229	66.9	11.1	242	55.9	7.7	229	61.9	10.4	242	51.6	7.2	1503	57.31	12.61	1163	50.68	10.94
** 15 to < 16 years**	311	75.6	11.4	377	60.6	7.7	311	70.0	10.7	377	56.0	7.2	957	59.78	14.38	772	52.32	11.59
** 16 to < 17 years**	220	77.9	10.8	287	61.9	8.1	220	72.2	10.1	287	57.1	7.6	605	62.66	12.57	552	54.13	11.58
**Urea (mmol/L)**																		
** 1 to < 17 years**	5413	4.44	0.98	5250	4.10	0.94	5413	4.50	1.00	5250	4.15	0.96	71,486	4.35	1.33	50,935	4.15	1.32

*PRINCE* Pediatric Reference Intervals in China, *LIS* Laboratory Information System, *SD* standard deviation

### Potential healthy population obtained by indirect sampling techniques

Creatinine and urea in the LIS data were divided into three clusters for each age and sex subgroup. The density curves of clusters partitioned by GMM and SOM are shown in Supplement Fig. 5 and Supplement Fig. 6, respectively. Corresponding distribution parameters are shown in Supplement Table 1 (GMM) and Supplement Table 2 (SOM). The middle density curve was identified as the distribution of potential healthy individuals that had normal levels of analytes. For GMM, children with normal creatinine accounted for 55% of the total (67,616/123,105), while children with normal urea accounted for 62% (75,489/122,421). For SOM, the percentages of potential healthy population were 18% (22,410/123,105) and 22% (26,513/122,421) for creatinine and urea, respectively, which appeared to be stricter than GMM in the partitioning of reference individuals.

### Reference distributions acquired by direct and indirect sampling techniques

**Fig. 1:**
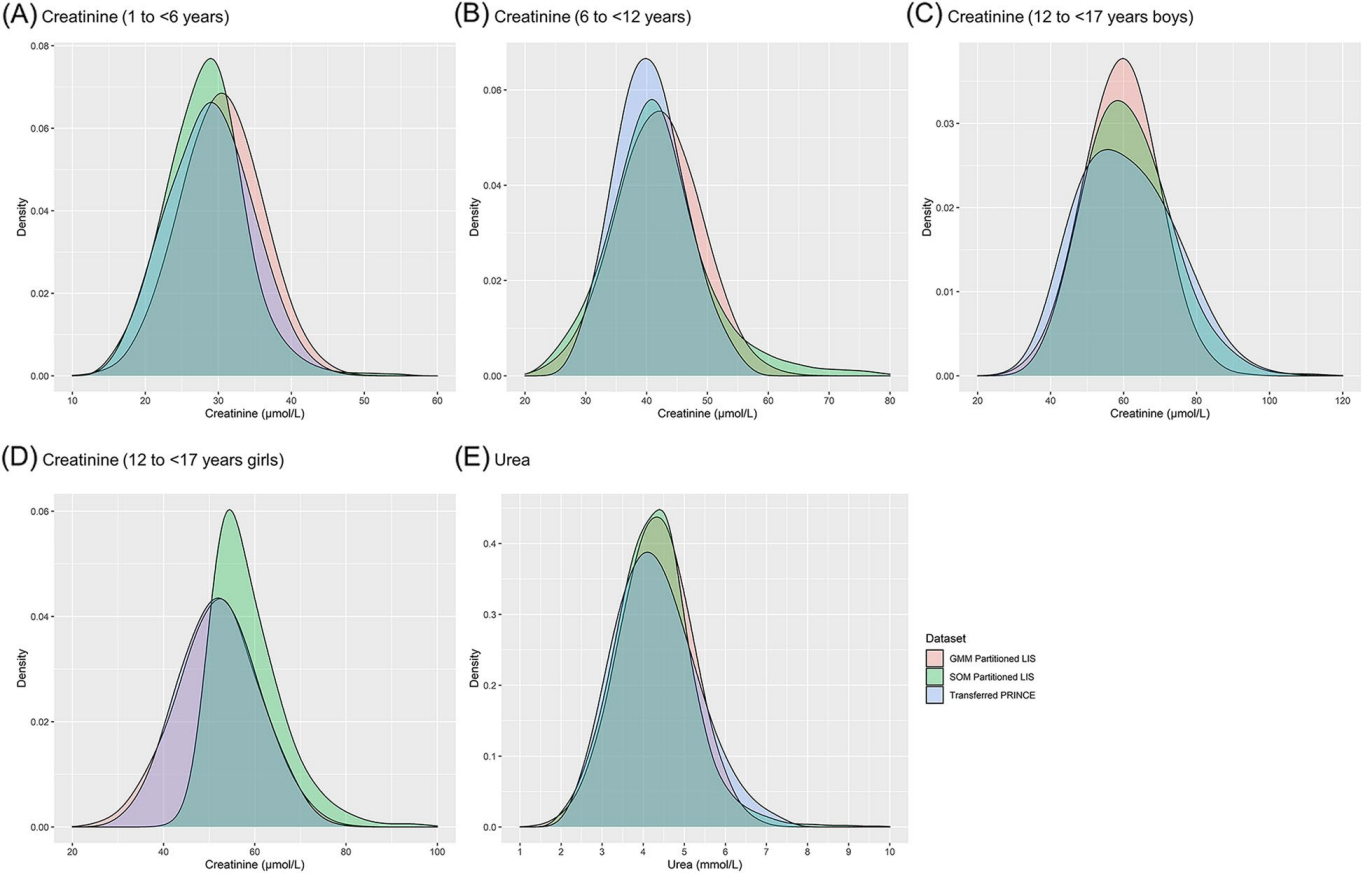
Reference distributions of creatinine and urea acquired by direct and two indirect sampling techniques. **A** Reference distribution of creatinine for children aged 1 to < 6 years. **B** Reference distribution of creatinine for children aged 6 to < 12 years. **C** Reference distribution of creatinine for boys aged 12 to < 17 years. **D** Reference distribution of creatinine for girls aged 12 to < 17 years. **E** Reference distribution of urea for children aged 1 to < 17 years. PRINCE: Pediatric Reference Intervals in China; LIS: Laboratory Information System; GMM: Gaussian Mixture Model; SOM: Self-Organizing Map

The probability density diagrams of creatinine and urea based on the transferred PRINCE data and the potential healthy populations partitioned by GMM and SOM from the LIS data are shown in Fig. 1. Since Gaussian fitting is the main procedure of GMM, the distributions of creatinine and urea shaped closer to normality, compared to direct sampling technique and SOM. Distribution parameters of the three methods are shown in Table 2.

**Table 2:** Reference distributions of creatinine and urea acquired by direct and two indirect sampling techniques

Age Group	n	Median	Mean	SD	Skewness	Kurtosis
Creatinine (μmol/L)						
1 to < 6 years						
Transferred PRINCE	3167	29.2	28.9	5.6	0.10	-0.33
GMM Partitioned LIS	33,692	30.4	30.4	5.7	-0.00	-0.01
SOM Partitioned LIS	10,854	28.2	28.2	5.5	0.54	1.88
6 to < 12 years						
Transferred PRINCE	4469	40.4	40.6	5.6	0.23	-0.28
GMM Partitioned LIS	26,400	41.9	41.9	7.0	0.01	0.00
SOM Partitioned LIS	6168	41.1	42.0	8.6	1.13	2.92
12 to < 17 years boys						
Transferred PRINCE	1455	60.1	60.8	12.7	0.37	-0.36
GMM Partitioned LIS	3826	59.4	59.3	10.0	0.01	-0.02
SOM Partitioned LIS	2819	60.4	61.5	11.7	0.57	0.62
12 to < 17 years girls						
Transferred PRINCE	1594	51.6	52.2	8.3	0.08	-0.30
GMM Partitioned LIS	3698	52.1	52.0	9.0	-0.01	-0.01
SOM Partitioned LIS	2569	56.8	58.5	7.8	1.31	2.67
Urea (mmol/L)						
1 to < 17 years						
Transferred PRINCE	10,663	4.25	4.33	0.99	0.43	-0.06
GMM Partitioned LIS	75,489	4.31	4.30	0.90	-0.01	-0.01
SOM Partitioned LIS	26,513	4.25	4.27	0.96	0.70	2.19

*PRINCE* Pediatric Reference Intervals in China, *LIS* Laboratory Information System, *GMM* Gaussian Mixture Model, *SOM* Self-Organizing Map, *SD* standard deviation

**Fig. 2:**
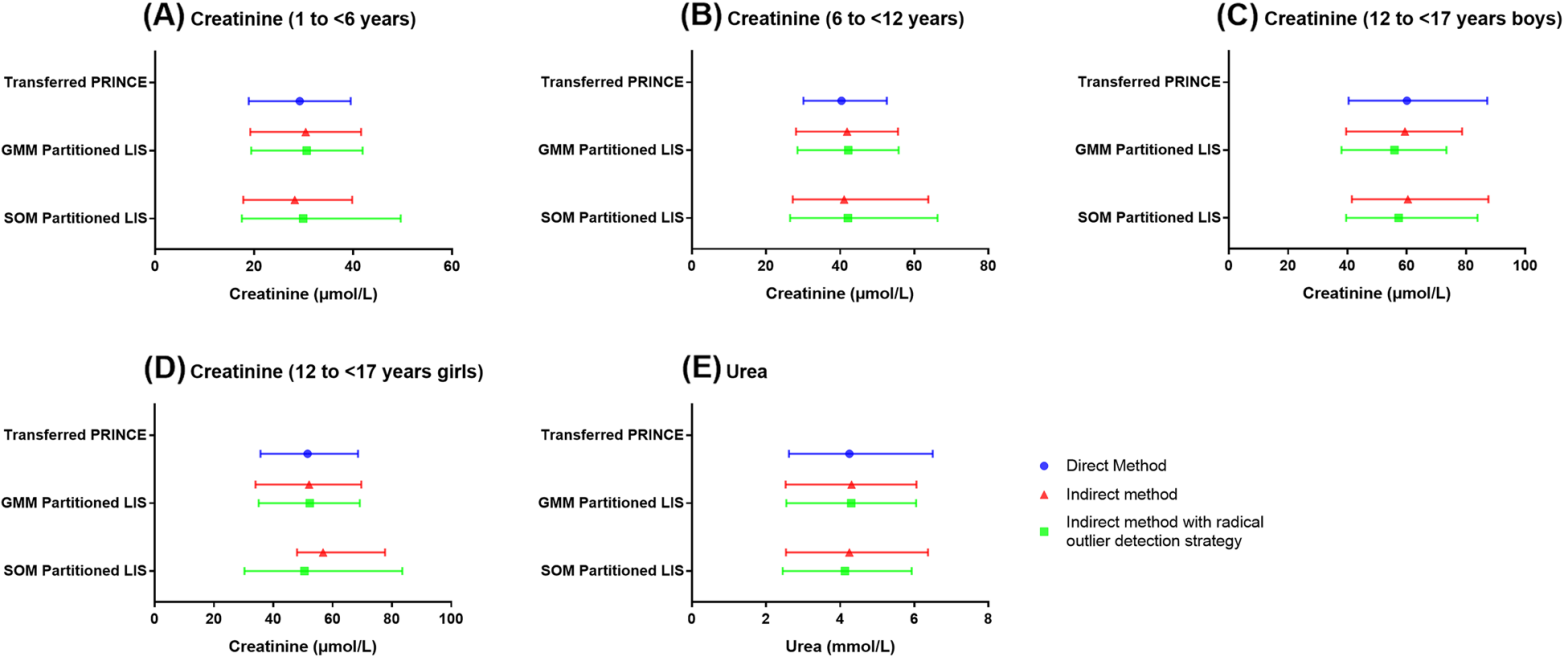
Reference intervals of creatinine and urea acquired by direct and two indirect sampling techniques. **A** Reference intervals of creatinine for children aged 1 to < 6 years. **B** Reference intervals of creatinine for children aged 6 to < 12 years. **C** Reference intervals of creatinine for boys aged 12 to < 17 years. **D** Reference intervals of creatinine for girls aged 12 to < 17 years. **E** Reference intervals of urea for children aged 1 to < 17 years. PRINCE: Pediatric Reference Intervals in China; LIS: Laboratory Information System; GMM: Gaussian Mixture Model; SOM: Self-Organizing Map

RIs of creatinine and urea established by direct and indirect sampling techniques are shown in Fig. 2 and Table 3. In subgroups of 1 to < 6 years and 12 to < 17 years girls where distributions of creatinine for direct samples were less skewed (skewness ≤ 0.10), the GMM partitioned LIS data presented similar RIs to the transferred PRICNE data. However, in subgroup of 12 to < 17 years boys where distribution of creatinine was away from normality (skewness was 0.37), SOM showed more advantages in RI calculation. As the distribution was right-tailed, GMM tended to underestimate, especially the upper limit of RI. Similar phenomenon was also observed for urea (skewness was 0.43).

**Table 3:** Reference intervals of creatinine and urea established by direct and two indirect sampling techniques

Age Groups	Transferred PRINCE		GMM Partitioned LIS		SOM Partitioned LIS	
	**LL (90% CI)**	**UL (90% CI)**	**LL (90% CI)**	**UL (90% CI)**	**LL (90% CI)**	**UL (90% CI)**
**Creatinine (μmol/L)**						
**1 to < 6 years**	18.9 (17.9, 18.9)	39.5 (39.5, 40.4)	19.2 (19.1, 19.3)	41.6 (41.5, 41.8)	17.8 (17.6, 18.0)	39.8 (39.4, 40.2)
**6 to < 12 years**	30.1 (30.1, 30.1)	52.6 (51.6, 52.6)	28.1 (27.9, 28.3)	55.6 (55.4, 55.7)	27.2 (27.0, 27.7)	63.8 (62.6, 65.1)
**12 to < 17 years boys**	40.4 (39.5, 41.3)	87.2 (85.3, 89.1)	39.6 (38.9, 40.3)	78.7 (78.1, 79.7)	41.5 (40.8, 42.2)	87.6 (85.5, 88.7)
**12 to < 17 years girls**	35.7 (34.8, 37.6)	68.6 (67.6, 69.4)	34.0 (33.4, 34.6)	69.7 (69.1, 70.7)	48.0 (47.8, 48.2)	77.7 (76.7, 79.2)
**Urea (mmol/L)**						
**1 to < 17 years**	2.62 (2.62, 2.62)	6.50 (6.50, 6.60)	2.53 (2.52, 2.55)	6.06 (6.05, 6.07)	2.54 (2.51, 2.56)	6.37 (6.31, 6.42)

*PRINCE* Pediatric Reference Intervals in China, *LIS* Laboratory Information System, *GMM* Gaussian Mixture Model, *SOM* Self-Organizing Map, *LL* lower limit, *UL* upper limit, *CI* confidence interval

### Sensitivity analysis

In sensitivity analysis, a more radical outlier detection strategy for the LIS data was used, and 116,144 children who had outliers in neither creatinine, urea, nor uric acid were included for indirect sampling. Corresponding RIs established by GMM and SOM are shown in Table 4. Compared with RIs presented in Table 3, SOM displayed higher sensitivity to outlier detection strategy than GMM. Nevertheless, regardless of which set of outliers were excluded, deviations existed in RIs based on direct and indirect sampling techniques, with bias ratio > 0.375 in most cases (Table 5).

**Table 4:** Reference intervals of creatinine and urea established by GMM and SOM with more radical outlier detection strategy

Age Groups	GMM Partitioned LIS		SOM Partitioned LIS	
	**LL (90% CI)**	**UL (90% CI)**	**LL (90% CI)**	**UL (90% CI)**
**Creatinine (mmol/L)**				
** 1 to < 6 years**	19.4 (19.3, 19.6)	41.9 (41.7, 42.0)	17.5 (17.3, 17.7)	49.6 (49.2, 50.3)
** 6 to < 12 years**	28.5 (28.3, 28.6)	55.8 (55.6, 56.0)	26.5 (26.2, 26.7)	66.3 (65.5, 67.0)
** 12 to < 17 years boys**	38.0 (37.2, 38.5)	73.4 (72.9, 74.4)	39.6 (39.0, 40.1)	83.9 (82.6, 84.8)
** 12 to < 17 years girls**	35.1 (34.6, 35.6)	69.2 (68.9, 69.6)	30.3 (29.3, 31.2)	83.5 (80.6, 87.4)
**Urea (mmol/L)**				
** 1 to < 17 years**	2.55 (2.53, 2.56)	6.05 (6.03, 6.06)	2.45 (2.43, 2.47)	5.93 (5.91, 5.97)

*LIS* Laboratory Information System, *GMM* Gaussian Mixture Model, *SOM* Self-Organizing Map, *LL* lower limit, *UL* upper limit, *CI* confidence interval

**Table 5:** Bias ratio of reference intervals of creatinine and urea establihsed by direct and two indirect sampling techniques

Age Groups	GMM Partitioned LIS		SOM Partitioned LIS		GMM Partitioned LIS with radical outlier detection strategy		GMM Partitioned LIS with radical outlier detection strategy	
	**LL**	**UL**	**LL**	**UL**	**LL**	**UL**	**LL**	**UL**
**Creatinine (μmol/L)**								
** 1 to < 6 years**	0.06	0.40	-0.21	0.06	0.10	0.46	-0.27	1.92
** 6 to < 12 years**	-0.35	0.52	-0.51	1.95	-0.28	0.56	-0.63	2.39
** 12 to < 17 years boys**	-0.07	-0.71	0.09	0.03	-0.20	-1.16	-0.07	-0.28
** 12 to < 17 years girls**	-0.20	0.13	1.47	1.08	-0.07	0.07	-0.64	1.78
**Urea (mmol/L)**								
** 1 to < 17 years**	-0.09	-0.44	-0.08	-0.13	-0.07	-0.45	-0.17	-0.58

*LIS* Laboratory Information System, *GMM* Gaussian Mixture Model, *SOM* Self-Organizing Map, *LL* lower limit, *UL* upper limit

## Discussion

Establishing RIs in pediatric practice remains a clinical challenging conundrum. Blood sample collection from healthy voluntary children is often subject to ethical or practical constraints. Therefore, in the present study, we used the LIS data from Beijing Children’s Hospital to partition potential healthy individuals by two indirect sampling techniques (GMM and SOM). Then, we compared the reference distributions based on indirect samples with those based on direct samples of the PRINCE study. Although the density curves of the three populations showed a large overlap, differences were found among corresponding RIs. Moreover, SOM demonstrated more sensitivity than GMM for different strategies of outlier detection, in terms of potential healthy subgroup division and RI establishment.

Generally, the accuracy of RI is largely dependent on the definition and recruitment of healthy individuals. The PRINCE study, as the first national initiative to develop pediatric RIs and improve laboratory test interpretation for the pediatric population in China, recruited reference individuals from communities or schools using pre-specified eligibility criteria [3]. By contrast, the LIS data contained all outpatients from a hospital, regardless of whether their disease might affect the laboratory test results or not (e.g., myopia, autism). Thus, algorithm such as GMM and SOM was needed to select potential healthy children for the establishment of RIs. Except for the disparity in sample sources, the difference between the PRINCE data and the LIS data also included: (1) the pre-analytical confounding factors such as fasting period, body temperature, or medication exposure were not recorded by the LIS data; (2) the sex, age, and region composition of the LIS data was not as strictly designed as the PRINCE data; (3) the specimens of the LIS data were fresh serums, compared with the frozen–thaw serums of the PRINCE data; (4) the different analyzers and reagents used might bring systematic errors in test results despite of transference. All above aspects might partially explain the deviations of reference distributions between the PRINCE data and the LIS data. Nevertheless, the identification of reference individuals (direct or indirect samples) was undoubtedly the main reason for the difference in RIs.

As for indirect sampling techniques, the number of clusters was set as two in some studies to represent healthy and unhealthy individuals, respectively [23, 24]. However, such approach is more suitable when the overall population is distributed skewedly, and unhealthy individuals mainly gather in the right or left tail of the density curve [25]. Neither the distribution of creatinine nor urea of the LIS data in the present study satisfied the above condition because both lower and upper levels of concentration could imply abnormality (Supplement Fig. 4). Thus, three clusters were specified to distinguish potential healthy individuals with moderate values of creatinine or urea from others.

In the GMM method, potential healthy population are partitioned based on the normality assumption, so that the reference distribution of indirect samples is apparently different from the original distribution of the LIS data. Assuming measures of both pathological and non-pathological children obeyed Gaussian distribution is an inherent limitation of GMM. Compared with GMM, SOM shows more advantages in the adaptability to data distribution. When the distribution of test results in healthy population is right-skewed, GMM may underestimate, especially the upper limit of RI, while SOM can give closer RI estimation to direct sampling technique. Moreover, SOM can simultaneously handle multiple related variables, and thus is particularly suitable for processing complex human physiological data. In our study, we explored the methodological application of SOM by taking renal function as an example, where 3 × 1 matrix of cluster grid was appropriate. If we focused on more health-related aspects, a more complicated matrix would be used.

Although SOM has its theoretical superiority, it encounters several limitations in practice. For instance, the stability of clustering may be affected by sample size. As displayed in Supplement Fig. 6, the separation of clusters by SOM was not consistent across four age groups of creatinine, which might be attributed to the relatively small sample sizes of 12 to < 17 years boys and girls. Because the boundary between children and adults blurs with age, adolescents may be diverted to general hospitals for medical treatment. Meanwhile, gender distinction is also required due to the appearance of secondary sexual characteristics after puberty. The above reasons together led to a sharp decrease in the sample size of children over 12 years of age, which further led to an unstable separation of clusters in Supplement Fig. 6C and 6D. Therefore, we recommend to use SOM in populations with sample size > 10,000. Beyond that, SOM has proven to be more susceptible to outliers, which suggests that outlier detection strategies should be carefully considered when using indirect sampling techniques to extract potential healthy individuals from the LIS data.

Another consideration is about the consistency and unbiasedness of GMM and SOM. As shown in Fig. 1 and Supplement Figs. 5 and 6, GMM tends to present more consistent results than SOM, but SOM tends to be closer to direct sampling technique. That being said, although GMM is more consistent, it may have some bias. Similar findings could also be drawn from Table 5, that is, among the five RIs acquired by GMM, only one had the bias ratio of both upper and lower limits less than 0.375, while three of the five RIs acquired by SOM reached the allowable threshold. In addition, potential healthy population partitioned by GMM tends to have smaller SD than SOM, and thus RIs based on GMM generally have narrower ranges than that based on SOM.

Furthermore, both GMM and SOM are unsupervised learning methods which are categorized as exploratory analysis. The process of data partitioning does not rely on any background knowledge or corresponding assumptions, but simply according to the similarity rules. In other words, the relationship of data in one cluster partitioned by GMM or SOM is as similar as possible, and the relationship of data among different clusters is as different as possible. Therefore, the interpretation of results based on indirect sampling techniques should carefully refer to professional knowledge and clinical implication.

To our knowledge, this study is the first attempt to directly compare indirect sampling techniques with classical direct sampling technique for RI establishment in Chinese children. Unlike most previous researches that could not explicitly determine whether the distribution of normal group acquired by indirect techniques was close enough to the actual healthy population, the major strength of our study is the availability to use the RPINCE data as a gold standard. Similar approach was also found in a recent paper, which made successful comparison between direct and four indirect methods, including Hoffmann, Bhattacharya, Arzideh, and Wosniok [22]. Our study reported another two methods, GMM and SOM, which could jointly guide the application of indirect sampling techniques in real world research.

The limitation of our study is that, pre-cleaning of the LIS data might not be vigorous enough, so that the distribution of the LIS data was biased from that of the PRINCE data, with regard to shifted peak or unmatched distribution width (Supplement Fig. 4). Although bias is inevitable due to the inherent difference between healthy children and outpatients, such bias might be reduced if more attempts were made in the first place. Furthermore, apart from data cleaning, pre-definition of the inclusion and exclusion criteria before data extraction is also important. Such issue has received little attention in current studies of indirect methods, and there is no existed guideline on how to set the eligibility criteria. Our team are conducting further research on advocating indirect sampling techniques based on the LIS data with pre-designed inclusion and exclusion criteria rather than only data cleaning.

## Conclusions

On all accounts, GMM and SOM could well identify potential healthy individuals from the LIS data, despite the reference distributions of indirect samples demonstrated certain difference from direct samples. Direct sampling technique is still a more accurate approach, while indirect sampling techniques can be used as a supplement when direct method is impractical or uneconomic in some circumstances. The performance of GMM is quite satisfactory, with consistent and stable estimation of RI. However, GMM relies on Gaussian fitting, and thus is not suitable for skewed data. By contrast, SOM shows advantages in the adaptability to data distribution, and is applicable for high-dimensional data. But it is susceptible to sample size and outlier detection strategy. It is imperative to develop more available indirect sampling techniques and to assess their feasibility by comparing the reference distribution with direct sampling techniques in future researches.

## Supplementary Information

**Additional file 1:** **Supplement Fig 1.** Age dependency of creatinine and urea by sex in the PRINCE data. **Supplement Fig 2.** Decision tree for age partitioning of creatinine in the PRINCE data. **Supplement Fig 3.** Data cleaning and outlier detection of the PRINCE data and the LIS data. **Supplement Fig 4.** Original distributions of the PRINCE data and the LIS data. **Supplement Fig 5.** Density curves of three clusters partitioned by GMM from the LIS data. **Supplement Fig 6.** Density curves of three clusters partitioned by SOM from the LIS data. **Supplement Table 1.** Distribution parameters of three clusters partitioned by GMM from the LIS data. **Supplement Table 2.** Distribution parameters of three clusters partitioned by SOM from the LIS data

## Data Availability

The datasets used and/or analysed during the current study are not publicly available due to project management requirements but are available from the corresponding author on reasonable request.

## Abbreviations

GMM: Gaussian Mixture Model
LIS: Laboratory Information System
PRINCE: Pediatric Reference Intervals in China
RI: Reference Interval
SOM: Self-Organizing Map
