# Supplementary material for: Comparison of reference distributions acquired by direct and indirect sampling techniques: exemplified with the Pediatric Reference Interval in China (PRINCE) study
Source: BMC Med Res Methodol. 2022 Apr 10;22:106. doi: 10.1186/s12874-022-01596-8 (PMC8996549; doi:10.1186/s12874-022-01596-8)
Supplement: Supplementary file 1 — Additional file 1: Supplement Fig 1. Age dependency of creatinine and urea by sex in the PRINCE data. Supplement Fig 2. Decision tree for age partitioning of creatinine in the PRINCE data. Supplement Fig 3. Data cleaning and outlier detection of the PRINCE data and the LIS data. Supplement Fig 4. Original distributions of the PRINCE data and the LIS data. Supplement Fig 5. Density curves of three clusters partitioned by GMM from the LIS data. Supplement Fig 6. Density curves of three clusters partitioned by SOM from the LIS data. Supplement Table 1. Distribution parameters of three clusters partitioned by GMM from the LIS data. Supplement Table 2. Distribution parameters of three clusters partitioned by SOM from the LIS data [file 12874_2022_1596_MOESM1_ESM.docx]

**Comparison of reference distributions acquired by direct and indirect sampling techniques: exemplified with the Pediatric Reference Interval in China (PRINCE) study**

Ruohua Yan, Kun Li, Yaqi Lv, Yaguang Peng, Nicholas Van Halm-Lutterodt, Wenqi Song, Xiaoxia Peng, Xin Ni

**Supplement Materials**

Supplement Fig. 1. Age dependency of creatinine and urea by sex in the PRINCE data

Supplement Fig. 2. Decision tree for age partitioning of creatinine in the PRINCE data

[Supplement](javascript:;) Fig. 3. Data cleaning and outlier detection of the PRINCE data and the LIS data

Supplement Fig. 4. Original distributions of the PRINCE data and the LIS data

[Supplement](javascript:;) Fig. 5. Density curves of three clusters partitioned by GMM from the LIS data

[Supplement](javascript:;) Fig. 6. Density curves of three clusters partitioned by SOM from the LIS data

Supplement Table 1. Distribution parameters of three clusters partitioned by GMM from the LIS data

Supplement Table 2. Distribution parameters of three clusters partitioned by SOM from the LIS data

**
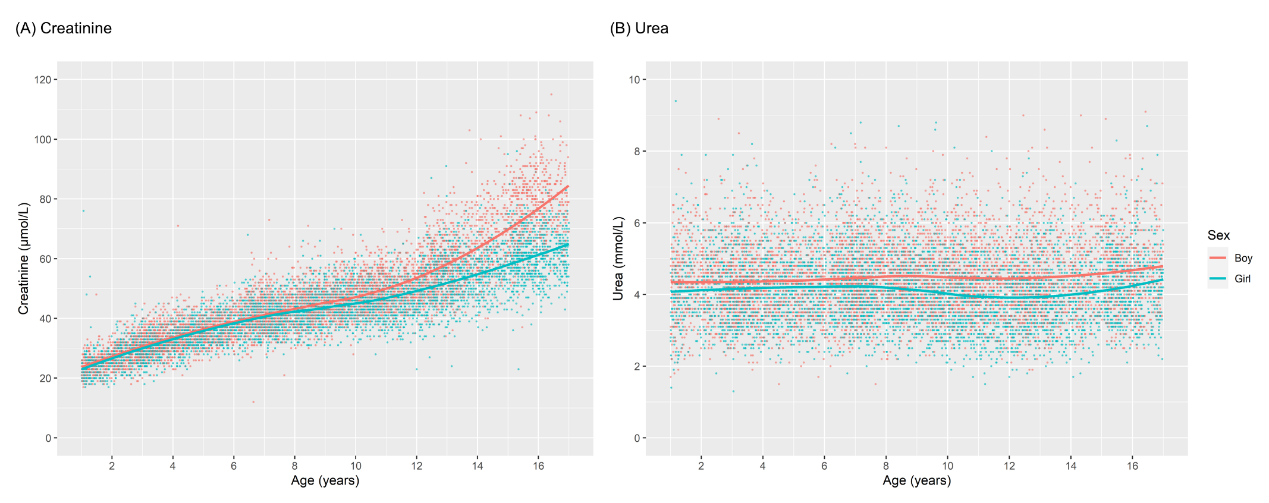
**

**Supplement Fig. 1. Age dependency of creatinine and urea by sex in the PRINCE data**

(A) Age dependency of creatinine by sex in the PRINCE data

(B) Age dependency of urea by sex in the PRINCE data

**PRINCE:** Pediatric Reference Intervals in China.

**
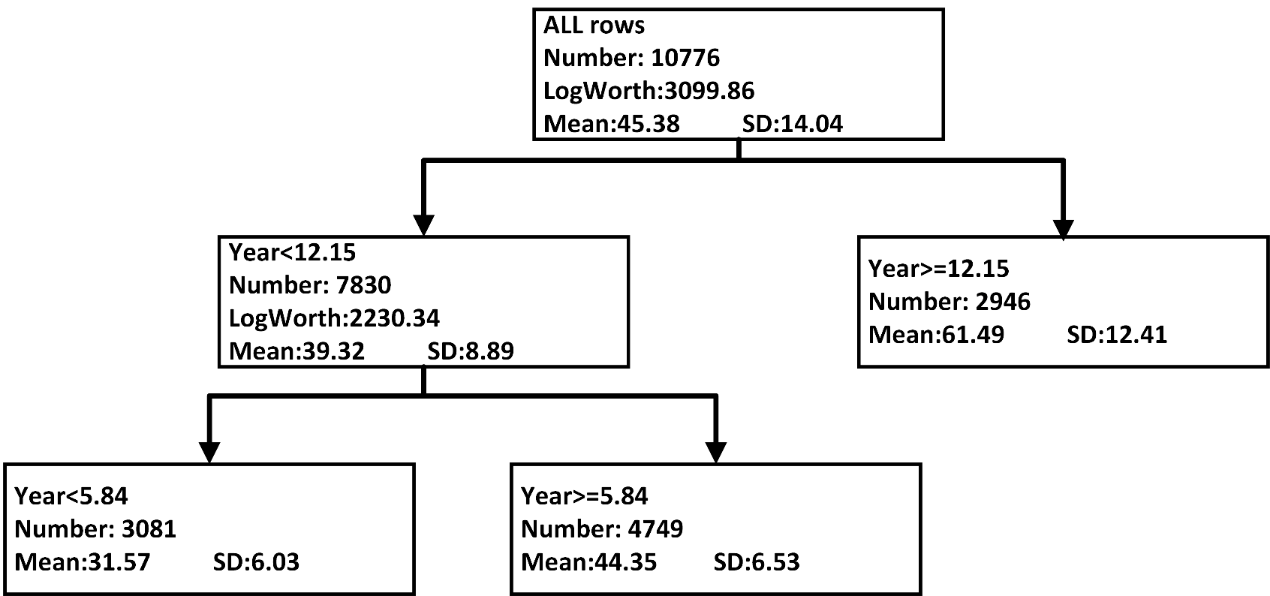
**

**Supplement Fig. 2. Decision tree for age partitioning of creatinine in the PRINCE data**

**PRINCE:** Pediatric Reference Intervals in China.


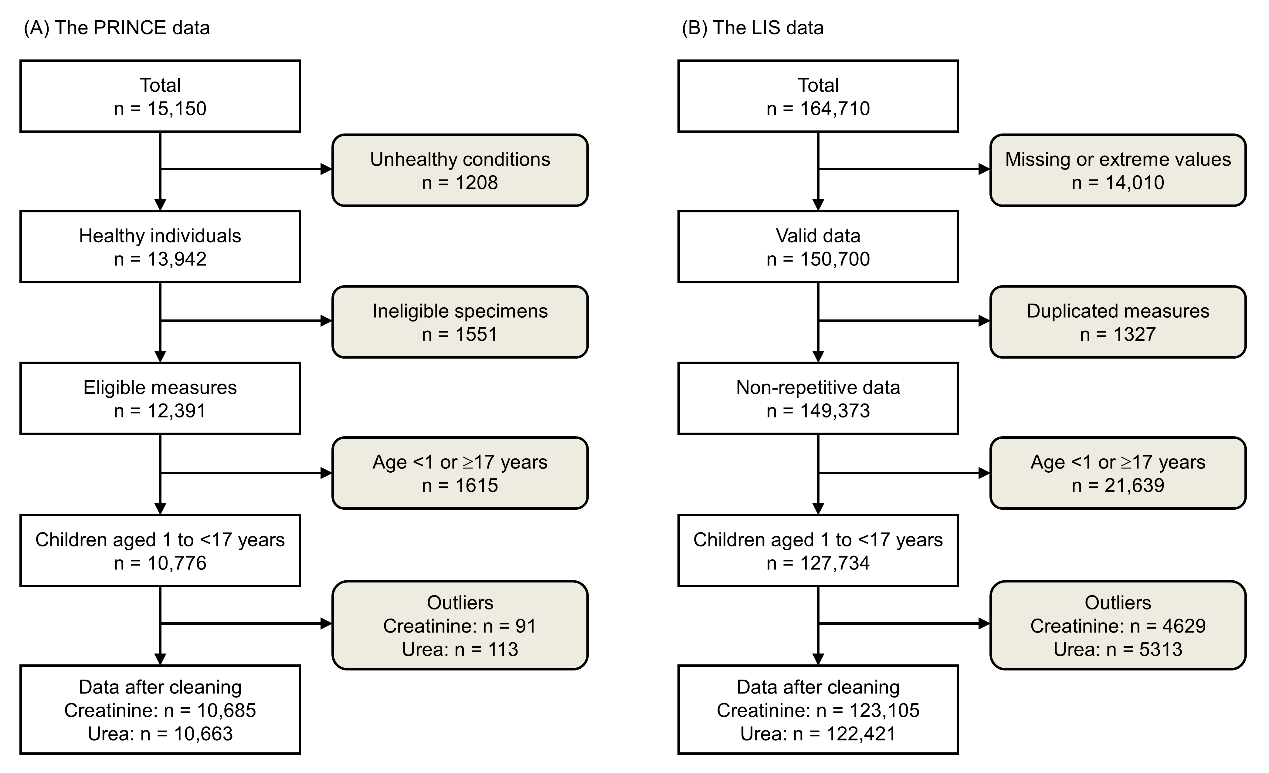


[**Supplement**](javascript:;) **Fig. 3. Data cleaning and outlier detection of the PRINCE data and the LIS data**

(A) Data cleaning and outlier detection of the PRINCE data

(B) Data cleaning and outlier detection of the LIS data

**PRINCE:** Pediatric Reference Intervals in China; **LIS:** Laboratory Information System.

**
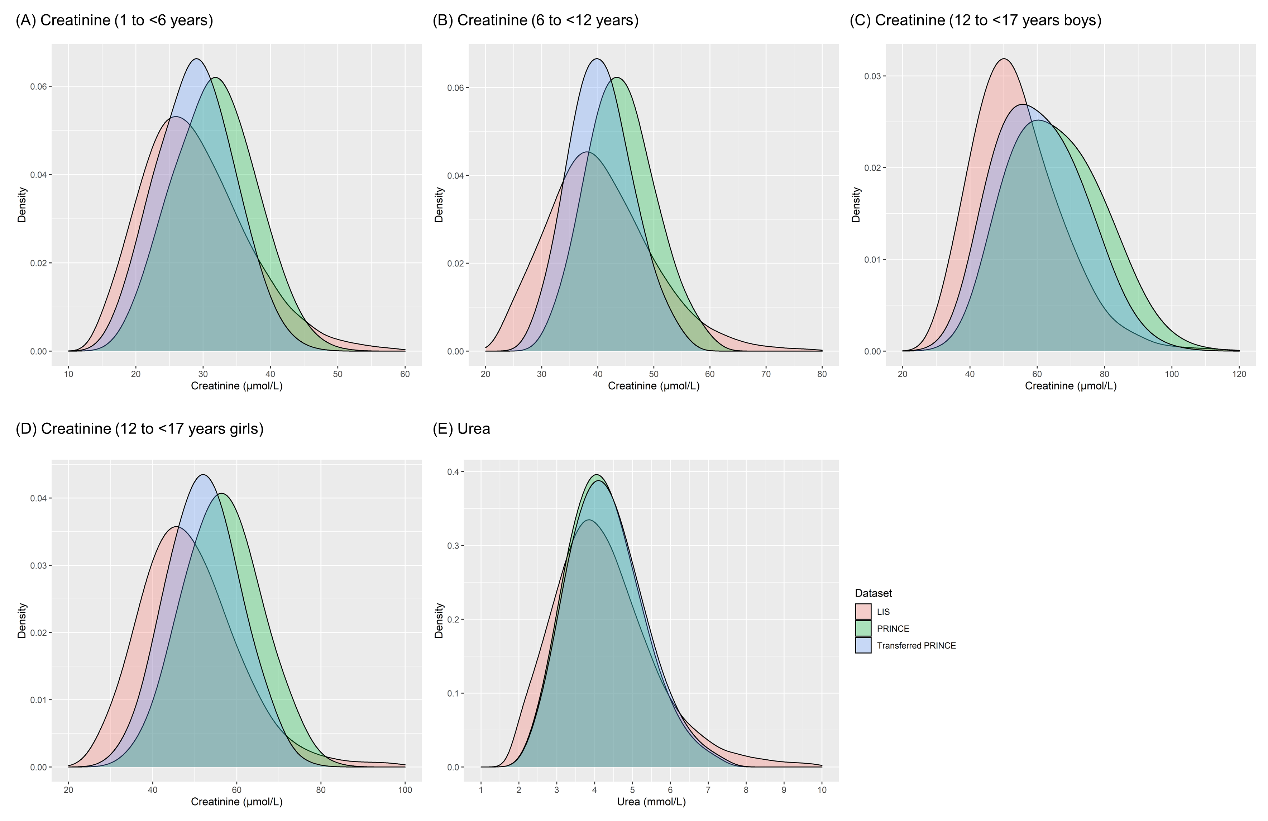
**

**Supplement Fig. 4. Original distributions of the PRINCE data and the LIS data**

(A) Original distribution of creatinine for children aged 1 to <6 years

(B) Original distribution of creatinine for children aged 6 to <12 years

(C) Original distribution of creatinine for boys aged 12 to <17 years

(D) Original distribution of creatinine for girls aged 12 to <17 years

(E) Original distribution of urea for children aged 1 to <17 years

**PRINCE:** Pediatric Reference Intervals in China; **LIS:** Laboratory Information System.

**
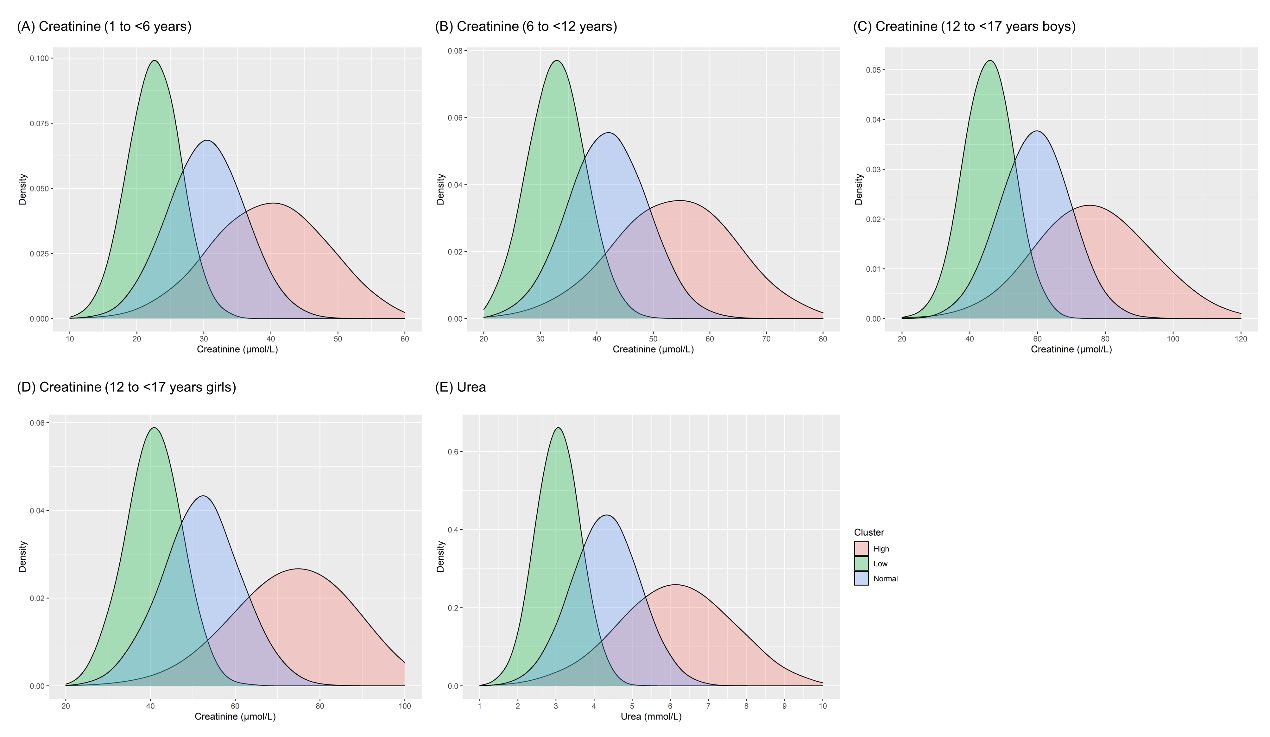
**

[**Supplement**](javascript:;) **Fig. 5. Density curves of three clusters partitioned by GMM from the LIS data**

(A) Density curves of creatinine for children aged 1 to <6 years

(B) Density curves of creatinine for children aged 6 to <12 years

(C) Density curves of creatinine for boys aged 12 to <17 years

(D) Density curves of creatinine for girls aged 12 to <17 years

(E) Density curves of urea for children aged 1 to <17 years

**LIS:** Laboratory Information System; **GMM:** Gaussian Mixture Model.

**
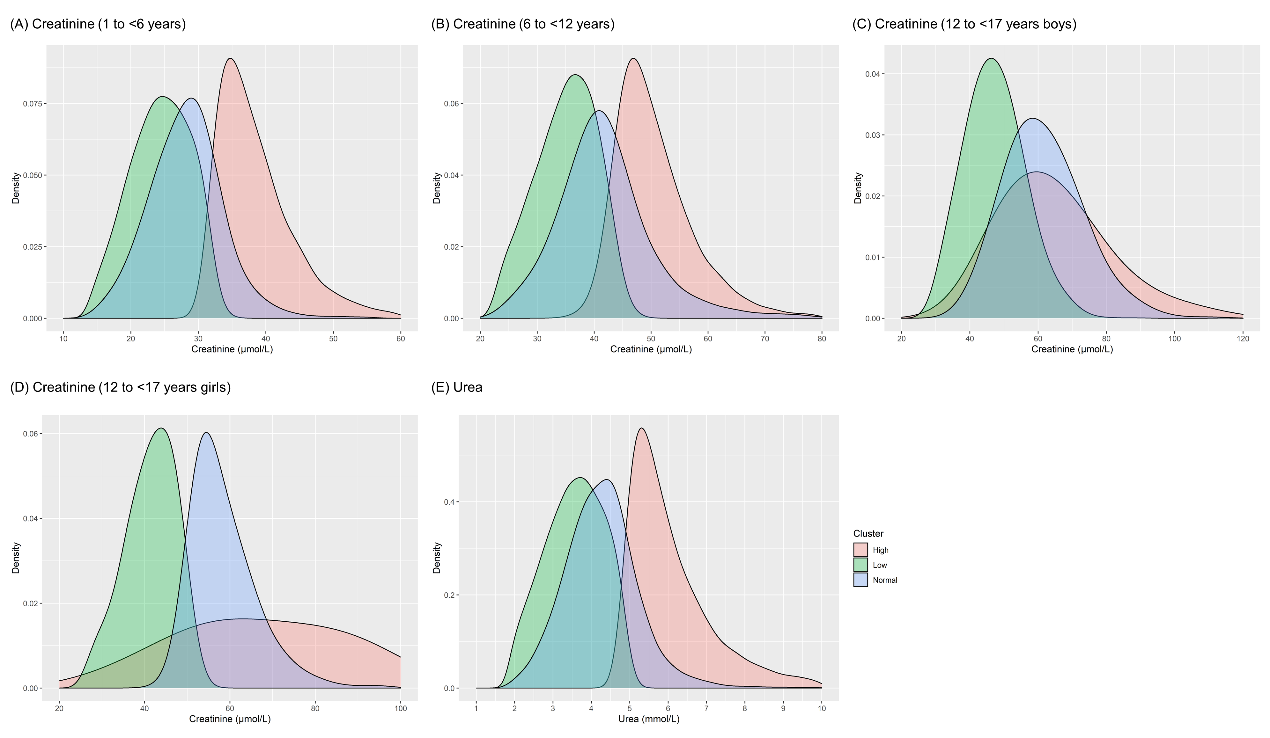
**

[**Supplement**](javascript:;) **Fig. 6. Density curves of three clusters partitioned by SOM from the LIS data**

(A) Density curves of creatinine for children aged 1 to <6 years

(B) Density curves of creatinine for children aged 6 to <12 years

(C) Density curves of creatinine for boys aged 12 to <17 years

(D) Density curves of creatinine for girls aged 12 to <17 years

(E) Density curves of urea for children aged 1 to <17 years

**LIS:** Laboratory Information System; **SOM:** Self-Organizing Map.

**Supplement Table 1. Distribution parameters of three clusters partitioned by GMM from the LIS data**

| **Age Groups** | **Cluster** | **Lambda (**λ**)** | **Mu (**µ**)** | **Sigma (**σ**)** |
| --- | --- | --- | --- | --- |
| **Creatinine (mmol/L)** |  |  |  |  |
| **1 to <6 years** | Low | 0.34 | 22.6 | 3.9 |
|  | Normal | 0.53 | 30.4 | 5.7 |
|  | High | 0.12 | 39.9 | 8.6 |
| **6 to <12 years** | Low | 0.30 | 32.8 | 5.0 |
|  | Normal | 0.59 | 41.9 | 7.0 |
|  | High | 0.12 | 53.8 | 10.8 |
| **12 to <17 years boys** | Low | 0.46 | 45.6 | 7.2 |
|  | Normal | 0.47 | 59.1 | 10.2 |
|  | High | 0.08 | 77.1 | 15.7 |
| **12 to <17 years girls** | Low | 0.38 | 40.7 | 6.5 |
|  | Normal | 0.57 | 52.2 | 8.9 |
|  | High | 0.05 | 74.1 | 12.4 |
| **Urea (mmol/L)** |  |  |  |  |
| **1 to <17 years** | Low | 0.24 | 3.06 | 0.59 |
|  | Normal | 0.62 | 4.31 | 0.90 |
|  | High | 0.14 | 6.15 | 1.51 |

**LIS:** Laboratory Information System; **GMM:** Gaussian Mixture Model.

[**Supplement**](javascript:;) **Table 2. Distribution parameters of three clusters partitioned by SOM from the LIS data**

| **Age Groups** | **Cluster** | **Lambda (**λ**)** | **Mu (**µ**)** | **Sigma (**σ**)** |
| --- | --- | --- | --- | --- |
| **Creatinine (mmol/L)** |  |  |  |  |
| **1 to <6 years** | Low | 0.56 | 24.9 | 4.5 |
|  | Normal | 0.17 | 28.2 | 5.5 |
|  | High | 0.27 | 38.6 | 5.7 |
| **6 to <12 years** | Low | 0.57 | 35.6 | 5.4 |
|  | Normal | 0.14 | 42.0 | 8.6 |
|  | High | 0.29 | 50.7 | 7.0 |
| **12 to <17 years boys** | Low | 0.54 | 48.0 | 8.8 |
|  | Normal | 0.34 | 61.5 | 11.7 |
|  | High | 0.12 | 64.6 | 16.1 |
| **12 to <17 years girls** | Low | 0.57 | 41.8 | 5.9 |
|  | Normal | 0.39 | 58.5 | 7.8 |
|  | High | 0.03 | 67.2 | 18.6 |
| **Urea (mmol/L)** |  |  |  |  |
| **1 to <17 years** | Low | 0.56 | 3.64 | 0.77 |
|  | Normal | 0.22 | 4.27 | 0.96 |
|  | High | 0.22 | 6.06 | 1.06 |

**LIS:** Laboratory Information System; **SOM:** Self-Organizing Map.
